# Efficient Generation of CRISPR/Cas9-Mediated Homozygous/Biallelic *Medicago truncatula* Mutants Using a Hairy Root System

**DOI:** 10.3389/fpls.2020.00294

**Published:** 2020-03-24

**Authors:** Hailing Zhang, Yingping Cao, Huan Zhang, Yue Xu, Chuanen Zhou, Wenwen Liu, Ruifen Zhu, Chen Shang, Jikai Li, Zhongbao Shen, Siyi Guo, Zhubing Hu, Chunxiang Fu, Dequan Sun

**Affiliations:** ^1^Grass and Science Institute of Heilongjiang Academy of Agricultural Sciences, Harbin, China; ^2^Shandong Technology Innovation Center of Synthetic Biology, Key Laboratory of Biofuels, Qingdao Institute of Bioenergy and Bioprocess Technology, Chinese Academy of Sciences, Qingdao, China; ^3^Qingdao Tianyun Ecological Technology Co., Ltd., Qingdao, China; ^4^The Key Laboratory of Plant Development and Environmental Adaptation Biology, Ministry of Education, School of Life Sciences, Shandong University, Qingdao, China; ^5^Collaborative Innovation Center of Crop Stress Biology, Henan Province and Institute of Plant Stress Biology, Henan University, Kaifeng, China

**Keywords:** CRISPR/Cas9, genome editing, hairy root, homozygous/biallelic mutant, *Medicago truncatula*

## Abstract

In the process of acquiring mutants mediated by CRISPR/Cas9, plantlets are often regenerated from both mutated and non-mutated cells in a random manner, which increase the odds of chimeric mutated plant. In general, it’s necessary to infect more explants or grow to next generation for the need of generating more biallelic or homozygous mutants. In present study, an efficient way of obtaining biallelic or homozygous mutated lines via fast-growing hairy root system without increasing numbers of infected explants or prolonging sexual propagation generation is reported. The fast growing lateral branches of hair roots are originated deep within the parental root from a small number of founder cells at the periphery, and therefore were employed as a library that classify different editing types in different lateral branches in which the homozygous or biallelic lines were screened. Here, *MtPDS* was employed in a proof-of-concept experiment to evaluate the efficiency of genome editing with our hairy root system. Homozygous/biallelic mutations were found only 1 of the 20 lines in the 1^st^ generation hairy roots, and 8 lines randomly selected were cultured to obtain their branch roots, homozygous/biallelic mutations were found in 6 of the 8 lines in their branch roots. We also tested the method with *MtCOMT* gene and got the same result. All of the seedlings regenerated from the homozygous/biallelic hairy root mutation lines of *MtPDS* displayed albino phenotypes. The entire process from vector design to the recovery of plantlets with homozygous/biallelic mutations took approximately 4.5–6.5 months. The whole process could bring inspiration for efficiently generating homozygous/biallelic mutants through CRISPR/Cas9 system from the hairy root or root system of a chimeric mutated transformants, especially for the rare and endangered plants whose explants sources are very limited or the plants that lack of tissue culture and rapid propagation system.

## Introduction

The clustered regularly interspaced short palindromic repeats (CRISPR) and CRISPR-associated protein 9 (Cas9) system is being harnessed as a powerful tool for gene engineering in crop improvement (Xie et al., 2015; Shah et al., 2018). In the majority of cases, an integration construct comprising CRISPR-Cas9, gRNA expression cassettes and a resistance gene (*hph*, *bar*, *npt II*, etc.) is transferred into plants using *Agrobacterium tumefaciens*, and the transformants are obtained through a selection process. As long as the resistance gene sequence is integrated into the plant genome and is expressed normally, transformed plants, tissues or calli can survive selection and grow regardless of whether the target sequences are edited by the CRISPR-Cas9 system or not. This results in the recovery of plants containing both mutated and non-mutated cells in a random manner, and increases the odds of recovering chimeric mutated plants (Nishitani et al., 2016; Pan et al., 2016). For example, in a previous study in rice, it was found that a prolonged tissue culture period could increase the chance of inducing *de novo* mutations in non-mutated cells, but the authors found no evidence that a prolonged tissue culture period could increase the rate of recovery of homozygous or biallelic mutants (Mikami et al., 2015). When introducing the CRISPR/Cas9 construct into Arabidopsis using the floral-dip genetic transformation method, homozygous or biallelic mutations were found in T2 plants but not in T0 or T1 plants (Feng et al., 2014). This is because the gene modifications detected in T1 plants occurred mostly in somatic cells; in other words, the T1 plants were chimeric for mutations in the target gene, and it was necessary to isolate plants with homozygous or biallelic mutations in the next generation (Feng et al., 2014).

In addition to *A. tumefaciens*, *Agrobacterium rhizogenes*, which contains the Ri plasmid and induces hairy roots, is another useful system for generating transgenics. This system has some obvious advantages. First of all, the Ri T-DNA, which induces hairy roots, can be transferred into plant cells along with the Ti T-DNA carrying the target gene. Thus, the CRISPR/Cas9 and gRNA expression cassettes could be transferred into the plant together with the Ri T-DNA via *A. rhizogenes* transformation (Li et al., 2017; Nakayasu et al., 2018). Secondly, hairy roots have the ability of infinite proliferation, and it takes only a very short time, about 5–10 days, to obtain new lateral branches. More importantly, it is easier to induce hairy roots using *A. rhizogenes* than to generate transformants using *A. tumefaciens*. Therefore, an *A. rhizogenes*-mediated transformation system has been established in many plants (Shi et al., 2014; Mehrotra et al., 2015; Li et al., 2017; Huang et al., 2018; Hudzieczek et al., 2018). The fact that only a small number of founder cells at the periphery of the parental root could be inherited and transferred their genetic materials in new formed lateral branches, but not retain all the calli as the situation in rice (Gibbs and Coates, 2014). This made us realize that the fast-growing branches of hairy roots could be considered a library in which different editing types produced by the CRISPR/Cas9 genome-editing system can be classified in different lateral branches. It is very likely that homozygous and biallelic mutants could be rapidly isolated from the branches of a hairy root line with chimeric mutations in the target gene or from newly created mutations in the process of subculture. In addition, by sub-culturing the hairy roots, more branch roots were gained in which more homozygous and biallelic mutated branches could be efficiently identified, without the need for time-consuming sexual reproduction as that required in transgenic Arabidopsis plants using the floral dip method. In addition, most plant hairy roots can be regenerated into a complete, functional plant that retains the original features of the plant from seed, which allows assessing gene function at a whole plant level (Choi et al., 2004; Crane et al., 2006; Zhou et al., 2007; Jia et al., 2008; Subotic et al., 2009; Sun et al., 2013; Sharafi et al., 2014; Hou et al., 2014; Habibi et al., 2016; Hudzieczek et al., 2018). The advantages of the hairy root system combined with the fact that the CRISPR-Cas9 system is well-established and has been used to efficiently perform gene editing in many plants (Feng et al., 2014; Nishitani et al., 2016; Qi et al., 2016; Wang P. C. et al., 2018) inspired us to engineer a hairy root-based system to efficiently obtain homozygous and biallelic mutants.

To establish this system, we chose to use *Medicago truncatula*, which has been adopted as a model for the study of genetics and genomics of forage legumes and is particularly useful for the study of root endosymbiotic associations (Horvath et al., 2015; Dhanushkodi et al., 2018), as our research subject. Fertile true transgenic plants have been regenerated from *A. rhizogenes*-transformed hairy roots of *M. truncatula* (Crane et al., 2006). In a previous study, it was reported that the CRISPR-Cas9 system could function in *M. truncatula* when introduced both through *A. tumefaciens*-mediated leaf disc transformation (Meng et al., 2017) and *A. rhizogenes*-mediated hairy root induction (Michno et al., 2015). In the present study, we demonstrate a fast and efficient method to obtain homozygous and biallelic mutants based on the *M. truncatula* hairy root system. We used a tRNA-gRNA expression strategy, in which multiple gRNAs are interspaced with tRNAs; the tRNAs work as a potential transcriptional enhancers for RNA polymerase III, boosting transcription, and can be precisely excised *in vivo* by endogenous RNases, releasing mature gRNAs (Xie et al., 2015; Cermak et al., 2017). Since it results in a high frequency of mutagenesis (up to 100%) and the tRNA-processing system is ubiquitous, this strategy has been successfully applied to many types of organisms (Qi et al., 2016; Dong et al., 2017; Kirchner et al., 2017; Deaner et al., 2018; Hashimoto et al., 2018; Wang Z. et al., 2018). In present work, a few homozygous/biallelic mutants were detected in the 1^st^ round of hairy root culture produced after transformation, and then a large number of additional homozygous and biallelic mutants were rapidly isolated in branches subcultured from the transgenic hairy roots. Our results indicate that the numerous CRISPR/Cas9-mediated homozygous/biallelic mutants can be obtained in one month after two rounds of hairy root culture. This hairy root selection system can facilitate a highly efficient generation of homozygous/biallelic mutants for genome engineering of forage and other agriculturally important crops.

## Results

### Hairy Root Induction and Characterization of Roots From 1^st^ Round of Culture

**FIGURE 1:**
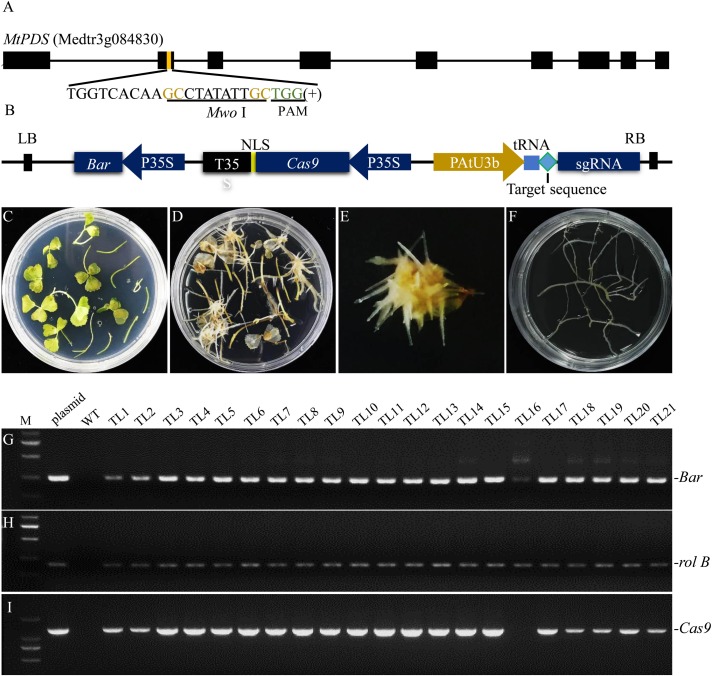
Identification of co-transformed hairy root lines of *M. truncatula* induced by *A. rhizogenes* strain LBA9402. **(A)** Schematic of the *MtPDS* gene and the selected target sequence. The nucleotides in brown font are part of the *Mwo*I recognition site, and the green font indicates the PAM. **(B)** Schematic illustration of the modified CRISPR/Cas9 construct used in this study. **(C)** The infected explants were transferred to selection medium containing 1.5 mg/L PPT. **(D)** Hairy roots began to appear from the leaf explants after inoculation with LBA9402 for about 10–15 days. **(E)** Approximately 10–15 hairy root lines were induced from the leaf blade. **(F)** Each line from the first generation of hairy roots was subcultured on a new plate to allow further growth. **(G–J)** PCR screening of DNA samples from the first generation. Untransformed wild type roots served as a negative control, and LBA9402 harboring the CRISPR/Cas9 plasmid was used as a positive control. The expected sizes of the *bar*, *Cas9, rolB*, and *virG* bands are indicated. TL, transgenic hairy root line.

*Agrobacterium rhizogenes* strain LBA9402 was employed to induce hairy roots of *M. truncatula* because this strain only has rifampicin resistance, which facilitates co-transformation with Ti T-DNAs carrying various other resistance genes. In addition, we found that of all the *A. rhizogenes* strains tested, LBA9402 exhibited the highest hairy root induction rate (Supplementary Table S1). Furthermore, we chose *MtPDS* as the target gene to examine the efficiency of genome editing in our hairy root system. One target sequence in the second exon of *MtPDS* containing a *Mwo*I restriction endonuclease cutting site next to the NGG region was selected for genotyping according to Ma et al. (2016; Figure 1A). Moreover, a tRNA-gRNA strategy was used for enhancing gRNA expression as described by Xie et al. (2015; Figure 1B). The CRISPR/Cas9 sequence and the gRNA expression cassette were cloned into a modified pYLCRISPR/Cas9 35S-B vector (Supplementary Figure S1). This vector was transferred into *M. truncatula* leaf explants together with the Ri T-DNA via LBA9402-mediated transformation. Hairy roots were induced from the leaf explants 10–15 days after inoculation (Figures 1C–E). Transgenic hairy root fragments approximately 2–4 cm length were cut from the leaves and then subcultured on SH solid medium (1^st^ round of culture) (Figure 1F). A fragment sampled from the main root without any lateral roots was used for genomic DNA extraction. Twenty-one transgenic hairy root lines (TLs) were randomly selected, and polymerase chain reaction (PCR) analysis showed that all of these lines contained *rolB*, which is a marker of hairy roots induced by *A. rhizogenes*. Of the hairy root lines, 95.5% were co-transformed with the modified binary vector pYLCRISPR/Cas9 35S-B carrying both *bar* and *Cas9*, none of them was amplified the *virG* product which excluded the *A. rhizogenes* contamination (Figures 1G–J).

### Isolation of Homozygous/Biallelic Mutants From the 1^st^ Round of Transgenic Hairy Roots Culture

**FIGURE 2:**
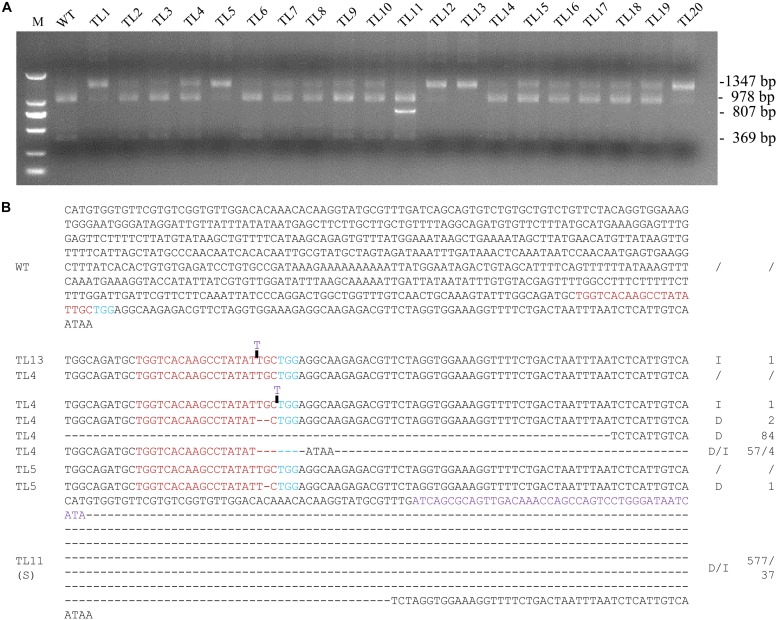
Screening for biallelic or homozygous mutated lines in the 1^st^ generation of hairy root lines. **(A)** PCR/restriction endonuclease assay to identify homozygous/biallelic lines for CRISPR/Cas9 genome edited mutations. Genotyping results for twenty 1^st^ generation hairy root lines are shown. The 1,347 bp band from amplification of *MtPDS* was cut into two bands 978 and 360 bp in size by the *Mwo*I restriction endonuclease. Lines 1–20, PCR products of co-transformed hairy root lines digested with *Mwo*I. Line 13 was homozygous for a mutation in *MtPDS*; the 1,347 bp band could not be digested. The PCR products in the other lanes were amplified from chimeric or heterozygous mutated hairy root lines; the 1,347 bp band was partially digested by *Mwo*I. M, 2 kb DNA marker. WT, digested PCR product amplified from the wild-type control. **(B)** Targeted genome editing on *MtPDS* gene in *M. truncatula* hairy roots. The sequence of *MtPDS* of untransformed hairy root and types of mutations in TL13, TL4, TL5, and the small band of TL11 in the first generation. Red bases indicate the target sequence; Blue bases indicate the PAM. The deletion is indicated by a dashed lines, and the inserted sequences are shown in blue letters, and the replaced sequences are shown in purple letters. d#, number of bases deleted from the target site. i#, number of bases inserted at the target site.

The region surrounding the targeted sequence in *MtPDS* was amplified by PCR from 20 co-transformed hairy root lines, and the PCR products were then subjected to *Mwo*I restriction endonuclease analysis. The 1,347 bp PCR product amplified from the wild-type control was completely digested into two fragments of 978 and 369 bp by *Mwo*I. In contrast, the PCR products amplified from all 20 co-transformed hairy root lines included bands at 1,347 bp that were not digested by *Mwo*I, implying that the *Mwo*I cleavage sites were disrupted by genome editing (Figure 2A). There was no digested bands in line 13, which suggesting that the target site of *MtPDS* in both alleles was edited (Figure 2A). Sequencing further revealed that line 13 was homozygous for a 1 bp insertion (T) (Figure 2B). Moreover, we selected three co-transformed hairy root lines 4, 5, and 11, which had digested bands, for further analysis. The sequence of the undigested PCR product amplified from line 4 showed that this line was a chimera with both the wild-type genotype and various types of mutations, including deletions and insertions (i1, d2, d84, and d57/i4), at the *MtPDS* target site (Figure 2B). In contrast, line 5 was a heterozygote containing the wild-type allele and a mutant allele with a 1 bp deletion (d1) in the target site (Figure 2B). There was an additional band smaller than the expected 1,347 bp in line 11 (Figure 2B). Unlike the 1,347 bp PCR fragment, the smaller fragment could not be digested by *Mwo*I (Supplementary Figure S2). Sequencing analysis revealed that the short sequence contained a 577 bp deletion, whereas the long sequence contained a 37 bp insertion (d577i37) in the target site (Figure 2B).

### Isolation of Homozygous/Biallelic Mutants From the 2^nd^ Round of Transgenic Hairy Roots Culture

**FIGURE 3:**
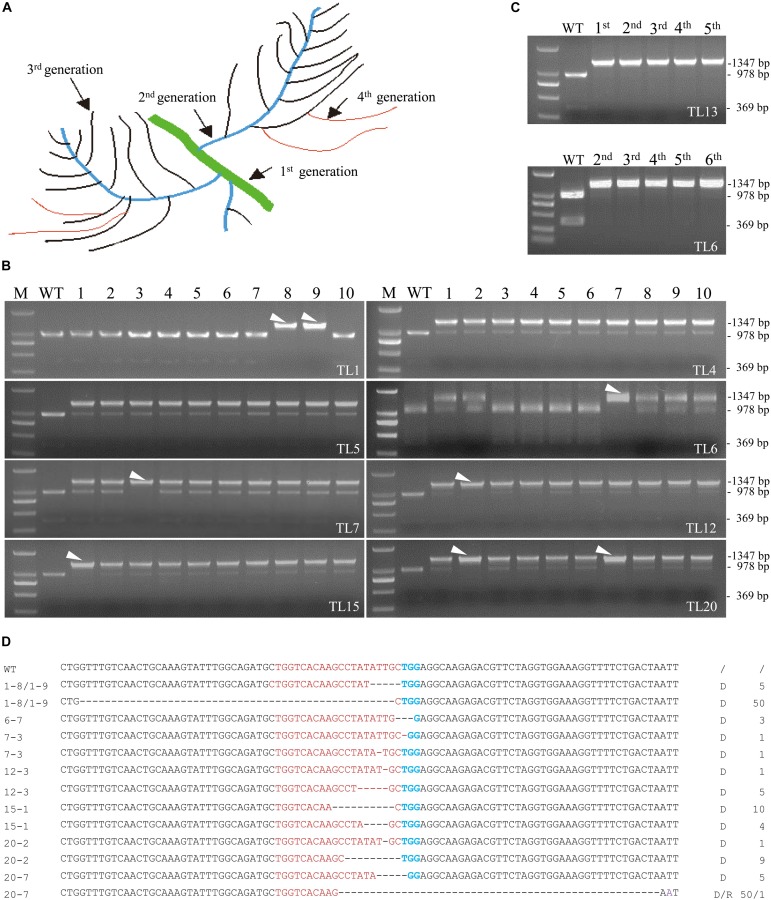
Screening of biallelic or homozygous mutation lines in the 2^nd^ generation of transgenic hairy root lines. **(A)** Schematic description of the different generations of hairy roots harvested. **(B)** Identification of eight biallelic or homozygous hairy root mutation lines in the 2^nd^ generation of lines 1, 4, 5, 6, 7, 12, 15, and 20. DNA samples from independent lines were analyzed for mutations using the PCR/restriction endonuclease assay. M, 2 kb DNA maker. WT, digested wild-type control; the 1,347 bp band corresponding to *MtPDS* was cut into two bands, 978 and 360 bp in size, by the *Mwo*I restriction endonuclease. Lanes 1–10, digested PCR products of independent transgenic hairy roots from the 2^nd^ generation of TL1, TL4, TL5, TL6, TL7, TL12, TL15, and TL20 with *Mwo*I. White arrowheads indicate the bands amplified from biallelic or homozygous hairy root mutation lines. **(C)** PCR/restriction endonuclease assay of five successive generations of line 6 (2^nd^ to 6th) and line 13 (1^st^ to 5th). In all lines of each generation, the 1,347 bp band could not be digested. **(D)** Types of mutations in the biallelic or homozygous hairy root mutation lines screened from the 2^nd^ generation of hairy roots. Red and green bases indicate the target sequence and PAM region, respectively. Deletions are indicated by dashed lines. d#, number of bases deleted from the target site. r#, number of bases replaced at the target site.

Although the 20 co-transformed hairy root lines from the 1^st^ round of culture were efficiently edited, all of them except line 13 were either heterozygous or chimeric (Figure 2A). Eight co-transformed hairy root lines from the 1^st^ round of culture including line 4, 5, 11, and 13 were subcultured for further investigation. Lateral roots produced from the primary roots in the 1^st^ round of culture were named 2^nd^ generation roots. Similarly, the lateral roots produced by the 2^nd^ generation roots were named the 3^rd^ generation roots, and so on (Figure 3A). Ten 2^nd^ generation hairy roots derived from each 1^st^ generation line were collected for PCR amplification and *Mwo*I restriction endonuclease analysis. At least one homozygous/biallelic root was recovered from the six out of eight lines selected based on the presence of a single indigestible band (Figure 3B). Sequencing data of the PCR products further revealed that, like line 13 in the 1^st^ generation, line 6 was also a homozygous mutant. However, line 6 contained a 3 bp deletion (d3) in the target site of *MtPDS* (Figure 3C). The homozygous mutation lines were evaluated for five successive generations, it was showed that their PCR products still could not be cut by *Mwo*I (Figures 3C,D). We continued to subcultured lines 6 and 13 and randomly selected their hairy roots to sequence (data not show) analysis, it revealed that the mutation type remained unchanged with continued subculturing (Supplementary Figure S5). In contrast, line 1 was found to be a biallelic mutant with a 50 bp (d50) deletion in one allele and a 5 bp (d5) deletion in the second allele of the target site (Figure 3D). Other types of mutation were found in the biallelic mutation lines: d1/d1 (1 bp deletions at different positions in the two alleles) in line 7–3, d1/d5 in line 12-3, d10/d4 in line 15-1, d1/d9 in line 20-2, and d5/d50 (r1) in line 20-7 (Figure 3D). Surprisingly, homozygous/biallelic mutations were not detected in the second generation roots of lines 4 and 5 (Figure 3B). Therefore, we examined the third and fourth generation roots of these lines. Unfortunately, no homozygous/biallelic mutations were found in the ten lines selected for analysis (Supplementary Figure S3).

In order to test the efficiency and stability of our experiment, we also designed a target site of another gene *MtCOMT* containing a *Bsl*I restriction endonuclease cutting sites at target site (Supplementary Figure S4A) and a tRNA-gRNA expression pattern was also used. Fifteen co-transformed hairy root lines of 1^st^ generation were selected for *Bsl*I digestion analysis (Supplementary Figure S4B). A total of 447 bp PCR product including the target site amplified from wide type *MtCOMT* was totally cut into two bands of 255 and 192 bp by *Bsl*I restriction endonucleases. One PCR product of the selected lines was found indigestible (Supplementary Figure S4B). Sequencing analysis indicated it was a homozygous mutant with 4-bp deletions at target sites of the two *MtCOMT* alleles (Supplementary Figure S4C). Four co-transformed lines, TL 1, TL 6, TL 9, and TL 11 were selected for 2^nd^ round selection. Ten 2^nd^ generation hairy roots derived from each 1^st^ generation line were collected for PCR amplification and *Bsl*I restriction endonuclease analysis. The homozygous/biallelic lines were found in three lines with the indigestible band as a symbol (Supplementary Figure S4D). Sequence results showed the mutation types of these homozygous/biallelic lines were as follows: d6/d3 in TL 1-8, d3 inTL6-4, d3/d117 in TL6-5, d4/d64 in TL11-2 (Supplementary Figure S4E). No homozygous/biallelic mutations were found in the 10 lines of TL9, sequence analysis showed that it was a chimeric mutant with at least three mutation types (Supplementary Figure S4E).

### Regeneration of Homozygous/Biallelic Mutants From Hairy Roots

**FIGURE 4:**
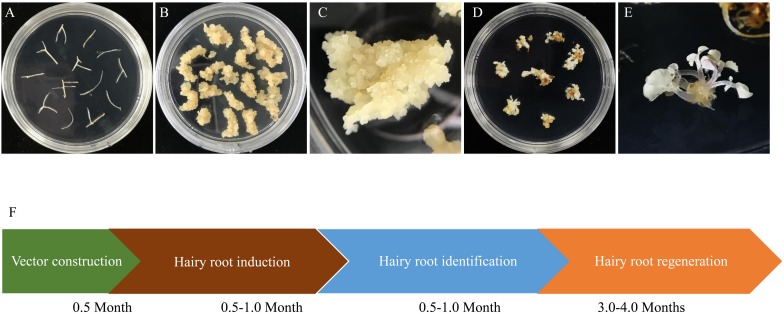
Regeneration of biallelic and homozygous mutated hairy root lines. **(A)** A represented hairy root line (TL13) with homogenous editing on *MtPDS* were cut into 1–2 cm lengths and transferred into callus induction medium. **(B)** Calli induced from hairy root fragments. **(C)** Close-up image of hairy root calli. **(D)** Plantlets with biallelic or homozygous mutations regenerated from hairy root calli. **(E)** Close-up image of plantlets with biallelic or homozygous mutations regenerated from hairy root calli. **(F)** Schematic of the process for obtaining biallelic and homozygous hairy root lines and the time required for each step. The entire process from vector design to the recovery of plantlets took approximately 4.5–6.5 months.

To obtain genome edited plantlets, calli were induced from the hairy roots with homozygous and biallelic mutations by culturing them on SH3a medium. Calli were then subcultured on MSBK medium for shoot regeneration as described by Jiang et al. (2019). We found that all regenerated plantlets derived from the homozygous/biallelic hairy root mutation lines displayed albino phenotypes as anticipated (Figure 4). The entire processes from target site design to recovery of regenerants took 4.5–6.5 months.

### Cas9 Expression Levels in Transgenic Hairy Roots

**FIGURE 5:**
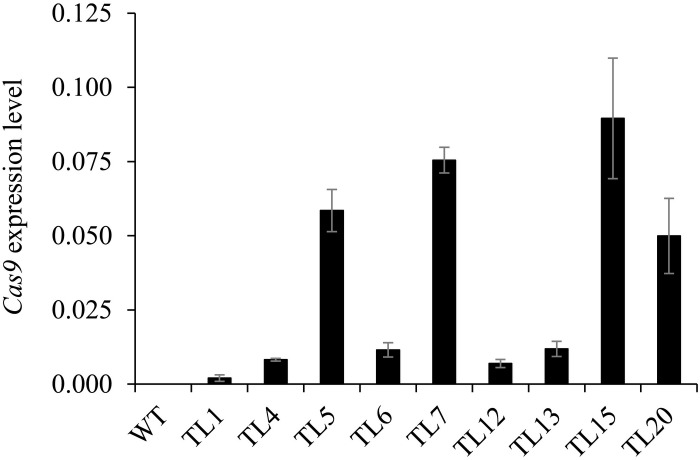
Expression levels of *Cas9* in co-transformed hairy root lines. Quantitative real-time PCR analysis of *Cas9* expression levels in 1^st^ generation hairy roots of nine lines. Root samples 10–15 days after inoculation were collected from untransformed wild type roots and co-transformed hairy root lines 1, 4, 5, 6, 7, 12, 13, 15, and 20. *M. truncatula Actin* (Medtr3g095530.2) was used as the reference for normalization. Value are mean ± SD (*n* = 3). WT, untransformed wild type roots.

The expression levels of Cas9 in nine selected hairy root lines from the 1^st^ round of culture were determined. We found great differences in Cas9 expression levels among the nine hairy root lines (Figure 5). Moreover, Cas9 expression level was not correlated with the efficiency of target gene editing in the co-transformed hairy roots. For example, a relatively low level of Cas9 expression was observed in the homozygous mutant (line 13) and the heterozygous and chimeric mutants (lines 1, 4, 6, and 12). In addition, a relatively high level of Cas9 expression was observed in some heterozygous and chimeric mutation lines (Figure 5).

## Discussion

Alfalfa (*Medicago sativa*) is the main source of vegetable protein in meat and milk production systems worldwide and is well known as “the queen of forage crops” because of its high protein content and high yield productivity (Stritzler et al., 2018). Moreover, this legume is also an ideal model for studying the interaction between rhizobia and plant roots (Crane et al., 2006). However, alfalfa has an autotetraploid genome, which limits the ability to identify gene functions (Meng et al., 2017). *M. truncatula* is a close relative of alfalfa and has several characteristics that make it a good model system, including a diploid genome that is easy to modify, a short life cycle, and a high level of natural diversity (Meng et al., 2017). Studies of *M. truncatula* could provide theoretical support for genetically modifying alfalfa. The new technology CRISPR/Cas9 is a powerful tool for generating targeted gene mutations and was previously applied successfully in *M. truncatula* (Meng et al., 2017). However, the rate of homozygous/biallelic mutant recovery was low, only 10% (Meng et al., 2017). In addition, the aseptic seedlings obtained by tissue culture process in previous study were difficult to develop roots, which reduced the recovery of homozygous/biallelic mutants as well (Crane et al., 2006).

Hairy roots of *M. truncatula* can be regenerated into complete plants that are easy to root (Crane et al., 2006). Therefore, in this paper, we used hairy roots as a system to implement CRISPR/Cas9 genome editing technology. Each hairy root line could be considered as being derived from an independent editing event. Hairy roots and their branches develop from only a small number of cells (Gibbs and Coates, 2014). That is to say, although a hairy root line is chimeric or heterozygous for a mutation, as long as a small number cells, which have the chance to form a branch, are homozygous/biallelic for the mutation, a homozygous/biallelic hairy root line can be isolated via culturing the branch roots. Since hairy roots are fast-growing, homozygous/biallelic lines can be efficiently isolated from the branches in a short period of time.

Previous studies have indicated that the recovery rate of homozygous/biallelic mutations in *MtPDS* generated by the CRISPR-Cas9 system is approximate 10%, which is higher than that observed in our hairy root lines in the 1^st^ round of culture (5%) (Meng et al., 2017). The different editing efficiencies could be the result of differences in GC content of the target sequences [55% in Meng et al. (2017)’s study vs 45% in our study] and/or secondary of structure between the target and gRNA (Ren et al., 2014; Liu et al., 2016). Although the homozygous/biallelic mutant recovery rate in our system was lower in the 1^st^ round of culture, when we selected homozygous/biallelic branch roots using a restriction enzyme digestion screening method, 75% of the hairy root lines (6 out of 8 lines) were found homozygous/biallelic in the second generation. Thus, the recovery rate dramatically increased from 5 to 75%, and this process only took 10–15 days. This result suggests that the lateral roots can potentially serve as a library containing abundant types of mutations for rapidly screening homozygous/biallelic mutants. Only the homozygous/biallelic branch roots were regenerated into complete plants in the subsequent culture, which avoided the unnecessary regeneration of plants without the desired target gene mutations.

In our study, we found that Cas9 expression levels have no correlationship with the recovery of homozygous/biallelic mutants. For example, homozygous mutant line 13 did not have higher Cas9 RNA levels than other heterozygous mutant or chimeric lines. Cas9 expression levels in first generation hairy roots of lines 1 and 6 were low, and biallelic mutations were detected in the second generation. In contrast, no homozygous or biallelic mutations were detected in the second generation hairy roots of line 5, in which Cas9 expression levels were relatively high (Figures 3, 5). One explanation for this might be that the RNA level is not the only factor that affects the activity of Cas9. Other possible causes, such as the abundance of Cas9 protein and the cell micro-environment, which were not investigated in the present study, would also influence Cas9 editing efficiency.

Another paradoxical phenomenon in our study is there were no biallelic or homozygous mutations were found in lines 4 and 5, even in its 3^rd^ and 4^th^ generation hairy roots lines (Figure 3B and Supplementary Figure S3). We ascribed this to the hairy root formation model and the sampling probability. The biallelic or homozygous lines could be detected only if two conditions were satisfied. Firstly, because hairy root can transfer only part but not all of its genetic materials to its branch roots, not like the situation in calli transformation method. Then, biallelic or homozygous lines can be identified only if cells carrying the mutation develop into a branch. In addition, the biallelic or homozygous branches were found only if they were selected for subculturing or subsequent analysis. Therefore, the possibility that biallelic or homozygous lines would have been detected by selecting more than 10 hairy roots from lines 4 and 5 cannot be ruled out. Moreover, we randomly sequenced the hairy root lines of line 5 of 3^rd^ and 4^th^ generations, there was still only one mutation type of 1 bp deletion at target site, no more new mutation type was detected (Supplementary Figure S3). This was consistent with the results in Arabidopsis, in which more than half (53%) of the mutations detected in T2 plants were not found at T1, but most T3 plants did not have new mutation (Feng et al., 2014). Likewise, in *M. truncatula*, it was found that mutations in T1 plant caused by CRISPR/Cas9 were transmitted through the germline rather than created *de novo* in T1 (Cermak et al., 2017). Therefore, it was suggested that taking more independent hairy roots of the 2^nd^ generation was more effective way to obtain biallelic or homozygous lines than subculturing more generations.

Taken together, we have reported an efficient method for obtaining biallelic or homozygous mutation lines using the fast-growing hairy root system. Because the plants regenerated from *M. truncatula* hairy roots are fertile, the mutated plants obtained by this strategy could allow assessment of gene function at the whole plant level. This strategy could be applied to other plants whose hairy roots can be regenerated into a normal plant, thus providing a different path for studying gene functions in non-model plants without a mature tissue culture regeneration system.

## Experimental Procedures

### Construction of CRISPR/Cas9 Expression Cassettes Vector

We replaced the fragment surrounded by *Bsa*I restriction sites and the *CCDB* gene in pYLCRISPR/Cas9 P35s-B (GenBank accession number: AI133729.1) (Ma et al., 2015) with sequences containing the AtU3b promoter and the sgRNA which were cloned from pYLsgRNA-AtU3b (GenBank accession number: KR029097.1), and there were also two new *Bsa*I restriction sites (Supplementary Figure S1A,B) between the promoter and sgRNA sequences. We named the new plasmid pYLCRISPR/Cas9 P35s-B-AtU3b and its annotated DNA sequences were in Supplementary Table S3. Then we used the simplified two-step method for vector construction with tRNA-gRNA cassettes described by Ma et al. (2016) and Xie et al. (2015) (Supplementary Figure S1C). The specific steps were as follows. Fragments were amplified from pGTR which contained the tRNA-sgRNA sequences and were synthesized by Sangon Biotech (Supplementary Figure S1D) (the primers used were listed in Supplementary Table S2), then the pYLCRISPR/Cas9 35s-B-AtU3b vector and amplified fragments were mixed for *Bsa*I enzyme digestion and T4 ligation (Ma et al., 2016; Xie et al., 2015). The vector containing the target sequence and the tRNA-gRNA expression cassette was transferred into *A. rhizogenes* strain LBA9402 for subsequent experiments.

### Selection of the gRNA Target Sequence

Sequences with 5′-N20NGG within either strand of the *MtPDS* (Medtr3g084830) were selected as candidate targets. According to Ma et al. (2016), sequences that having GC content lower than 40% were removed. And then we eliminate the candidate targets having four or more consecutive T nucleotides to avoid early transcriptional termination of AtU3b. Then, for convenient identification of the homozygous or biallelic mutation line, we detected the restriction enzyme cutting sites of the rest candidate sequences. The target sequence, TGGTCACAAGCCTATATTGC(TGG), having a *Mwo*I cutting sites close to the NGG, were selected as our target sequence.

### Hairy Root Induction by *A. rhizogenes* and Subculturing

Healthy leaves and petioles of aseptic seedlings of *M. truncatula* genotype R108 (Hoffmann et al., 1997) were employed for *A. rhizogenes* strain LBA9402 infection as describe by Crane et al. (2006). After 2 days of cocultivation in SH medium containing 0.196 mg l^–1^ acetosyringone (MDBio, Taiwan, China), the infected explants were transferred to selection medium, SH medium supplemented with 10 g l^–1^ sucrose, 1.5 mg l^–1^ phosphinothricin (PPT) (PhytoTechnology Laboratories) and 400 mg l^–1^ timentin (MDBio, Taiwan, China). Hairy roots were subcultured every 10–15 days onto fresh selection medium.

### Identification of Co-transformed Hairy Roots

The T0 generation hairy roots were cut from leaves and subcultured on new SH medium. The branches of the T0 generation hairy roots were named 1^st^ generation hairy roots. Similarly, branches of 1^st^ generation hairy roots were named 2^nd^ generation hairy roots, and so on. Hairy roots of different generations were collected. The *bar*, *Cas9*, and *rolB* genes were amplified using T5 direct PCR kit (plant) (Tsingke biotech, Beijing, China) with the primers listed in Supplementary Table S2. PCR was performed in a thermal cycler with the following program: 95°C for 5 min, 30 cycles of 95°C for 30 s, 55 for 30 s, and 72°C for 1.5 min, and 72°C for 10 min.

### PCR/Restriction Endonuclease Assay of Transgenic Hairy Root Mutations

Polymerase chain reaction amplification of a 1,347 bp region surrounding the target sequence was performed on selected co-transformed DNA samples using the primers listed in Supplementary Table S2. PCR was performed using T5 direct PCR kit (plant) (Tsingke biotech, Beijing, China) in a thermal cycler with the following program: 95°C for 5 min, 30 cycles of 95°C for 30 s, 55°C for 30 s, 72°C for 1.5 min, and 72°C for 10 min. The PCR products were then run on a 1% agarose gel, and the target bands were extracted using the Omega Gel Extraction Kit (Omega Bio-tek, GA, United States). Target gel extraction products (∼1,000 ng) were digested with 5 u *Mwo*I restriction enzyme at 60°C for 40 min. Digested DNA was then run on a 1% agarose gel in 1 × TBE for 20 min at 180 V, then imaged on a UV gel imager (Bio-Rad Laboratories, Shanghai, China). Gel bands that appeared to be undigested were then extracted using the Omega Gel Extraction Kit (Omega Bio-tek, GA, United States). And cloned into the pMD19-T easy vector (TaKaRa, Toyoto, Japan). Five to twenty clones for each line were sequenced.

### Regeneration of Hairy Roots

The homozygous and biallelic hairy root lines were cut into 1–2 cm lengths and transferred onto SH3a medium [N6 major salts, SH minor salts and SH vitamins, supplemented with 4.0 mg l^–1^ 2,4-D, 0.5 mg l^–1^ BAP, 3% (w/v) sucrose, 200 mg l^–1^ timentin (MDBio, Taiwan, China), and solidified with 0.75% (w/v) agar (Sangon Biotech, Shanghai, China)]. Induced calli were transferred onto MSBK medium [MS basal medium supplemented with 0.5 mg l^–1^ BAP, 1 mg l^–1^ kinetin, 3% (w/v) sucrose, 200 mg l^–1^ timentin (MDBio, Taiwan, China) and solidified with 0.75% (w/v) agar] for bud differentiation as described by Jiang et al. (2019).

### Cas9 Gene Expression Analysis via RT-qPCR

Total RNA was extracted from individual hairy roots of *M. truncatula* using Invitrogen Trizol Reagent (Invitrogen, CA, United States) according to manufacturer’s instructions. qRT-PCR was conducted on LightCycler 480 Detection System (Roche, Penzberg, Germany) using SYBR Premix Ex Taq (TaKaRa, Toyoto, Japan) as described by Hu et al. (2010). The primer sets used are listed in Supplementary Table S2. An *Actin* gene (Medtr3g095530.2) was used as an internal control to calibrate variation in cDNA concentration (Niu et al., 2015).

### Statistical Analysis

Samples collected from three technique replicates of each TL. The mean values were used for statistical analysis. Data from each trait were subjected to one-way analysis of variance (ANOVA). Standard derivations (SD) were provided in all figures and tables as appropriate.

## Data Availability Statement

All datasets generated for this study are included in the article/Supplementary Material.

## Author Contributions

HLZ and YC contributed equally to this work. HLZ, YC, CZ, DS, and CF designed the research. HLZ, YC, HZ, YX, WL, RZ, CS, and JL performed the experiments. ZS, SG, and ZH analyzed the data. YC and CF wrote the manuscript.

## Conflict of Interest

The authors declare that the research was conducted in the absence of any commercial or financial relationships that could be construed as a potential conflict of interest.
