# Supplementary material for: Efficient Generation of CRISPR/Cas9-Mediated Homozygous/Biallelic Medicago truncatula Mutants Using a Hairy Root System
Source: Front Plant Sci. 2020 Mar 24;11:294. doi: 10.3389/fpls.2020.00294 (PMC7105802; doi:10.3389/fpls.2020.00294)
Supplement: Supplementary file 1 [file Presentation_1.pdf]

Supporting information

Fig. S1

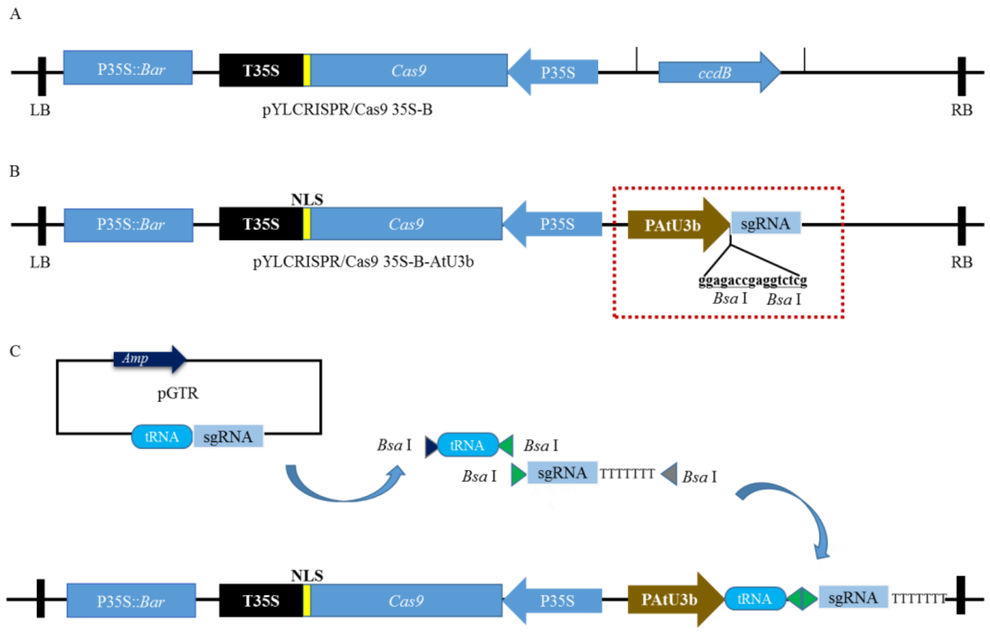

D

CGTTTTAGAGCTAGAAATAGCAAGTTAAAATAAGGCTAGTCCGTTATCAACTTGAAAAAGTGGCACCGAGTCGGTGCAACAAAGCAC
CAGTGGTCTAGTGGTAGAATAGTACCCTGCCACGGTACAGACCCGGGTCCGATTCCCGGCTGGTGCA

Figure S1 Schematic description of the modified pYLCRISPR/Cas9 35S-B system. (A) Schematic illustration of pYLCRISPR/Cas9 35S-B. The red box indicates the region that was modified in the present study. NLS, nuclear localization sequence. (B) Schematic illustration of the modified CRISPR/Cas9 construct used in the present study. The red box indicates the region that was modified in the present study. NLS, nuclear localization sequence. (C) Schematic illustration of the pGTR plasmid containing the tRNA and sgRNA scaffolds, and the process for constructing the expression vector comprising both the tRNA-gRNA expression cassette and Cas9. NLS, nuclear localization sequence.

Fig S2

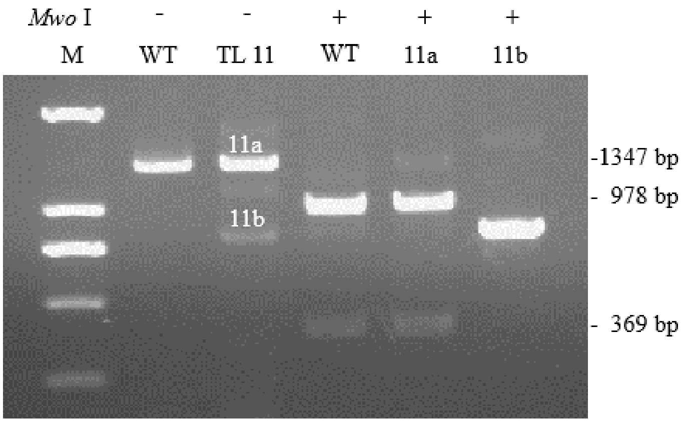

Figure S2 PCR/ restriction endonuclease assay to detect CRISPR/Cas9 genome edited mutations in the 1st generation of line 11. M, 2 kb DNA marker. -, undigested PCR products. +, digested PCR products. WT, PCR product of wild type hairy root of *M. truncatula*. TL11, PCR product of transgenic hairy root line 11. 11B, PCR product of the top band of transgenic hairy root line 11 purified by gel extraction kit. 11S, PCR product of the middle band of transgenic hairy root line 11 purified by gel extraction kit digested.

Fig S3

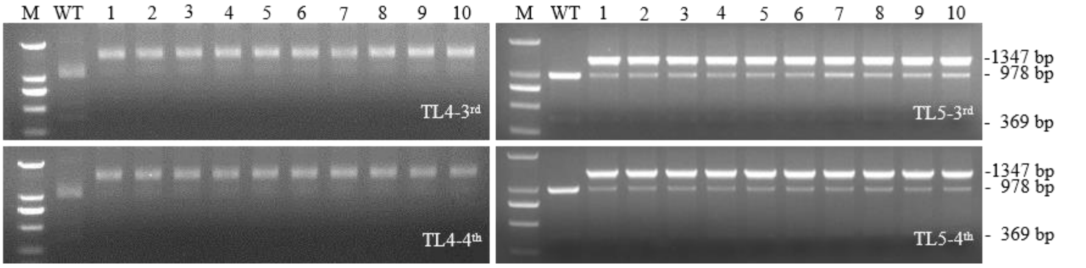

Figure S3 PCR/restriction endonuclease assay to identify CRISPR/Cas9 genome edited mutations in the 3rd and 4th generations of transgenic hairy root lines 4 and 5. PCR products amplified from transgenic hairy root line 4 and 5 digested with *Mwo* I. M, 2 kb DNA marker. WT, digested PCR product of wild type hairy root of *M. truncatula*; the 1,347 bp band corresponding to *MtPDS* was cut into two bands, 978 bp and 360 bp in size, by the *Mwo* I restriction endonuclease. TL4-3rd, the third generation of TL4. TL4-4th, the fourth generation of TL4. TL5-3rd, the third

generation of TL5. TL5-4th, the fourth generation of TL5. 1-10, digested PCR products of independent transgenic hairy roots from the 3rd and 4th generation of TL4 and TL5. No biallelic or homozygous hairy root mutation lines were found.

Fig. S4

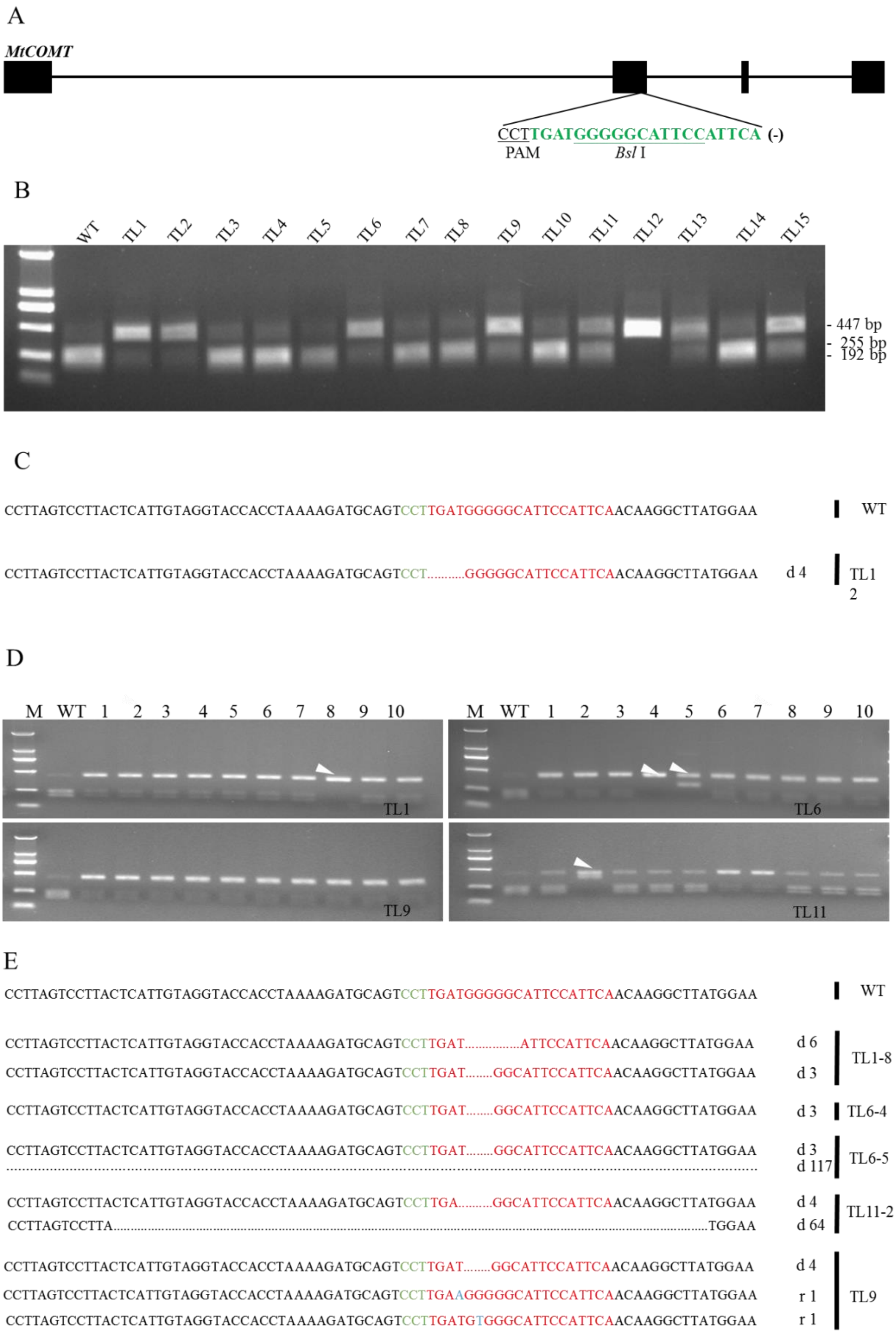

Figure S4 Screening for biallelic or homozygous mutated lines of *MtCOMT* in the 1st and 2nd generation of hairy root lines. (A) Schematic of the *MtCOMT* gene and the

selected target sequence. The nucleotides in green font were the target site in which the *Bsl* I recognition site were marked with blue lines, and the black font indicates the PAM. (B) PCR/restriction endonuclease assay to identify homozygous/biallelic lines for CRISPR/Cas9 genome edited mutations. Genotyping results for twenty 1st generation hairy root lines are shown. The 447 bp band from amplification of *MtCOMT* was cut into two bands 255 bp and 192 bp in size by the *Bsl* I restriction endonuclease. Lines 1-15, PCR products of co-transformed hairy root lines digested with *Bsl* I. Line 12 was biallelic for a mutation in *MtCOMT*; the 447 bp band could not be digested. The PCR products in the other lanes were amplified from chimeric or heterozygous mutated hairy root lines; the 447 bp band was partially digested by *Bsl* I. M, 2 kb DNA marker. WT, digested PCR product amplified from the wild-type control. (C) The sequence of *MtCOMT* of untransformed hairy root and types of mutations in TL12 in the first generation. Red bases indicate the target sequence; Green bases indicate the PAM (color figure online). The deletion is indicated by a dashed lines. d#, number of bases deleted from the target site. (D) Identification of five biallelic or homozygous hairy root mutation lines in the 2nd generation of lines 1, 2, 6, 9, and 15. DNA samples from independent lines were analyzed for mutations using the PCR/restriction endonuclease assay. M, 2 kb DNA maker. WT, digested wild-type control; the 447 bp band corresponding to *MtCOMT* was cut into two bands, 255 bp and 192 bp in size, by the *Bsl* I restriction endonuclease. 1-10, digested PCR products of transgenic hairy roots from the 2nd generation of TL1, TL2, TL6, TL9, and TL15 with *Bsl* I. White arrowheads indicate the bands amplified from biallelic or homozygous hairy root mutation lines. (E) Types of mutations in the biallelic or homozygous hairy root mutation lines screened from the 2nd generation of hairy roots and mutation types of 1st generation hairy root of TL9. Red and green bases indicate the target sequence and PAM region, respectively. Deletions are indicated by dashed lines. d#, number of bases deleted from the target site. r#, number of bases replaced at the target site.

Fig S5

GCAGATGCTGGTCACAAGCCTATATTTGCTGGAGGCAAGAGACGTTCTAGGTGGAAAGGTTTTCTGACTAATT i1 | TL13

GCAGATGCTGGTCACAAGCCTATATTG...........GAGGCAAGAGACGTTCTAGGTGGAAAGGTTTTCTGACTAATT d3 | TL6-7

Figure S5 Targeted genome editing on *MtPDS* gene of continuous subculture generations of the two homozygous mutations found in line 13 and line 6. The mutation types of the two homozygous mutant lines were unchanged in the continuous subculture generations.

Table S1 The hairy root induction efficiency using different *A. rhizogenes* strains

A. rhizogenes	Total number of explants	Induction efficiency
LBA9402	89	67.7%±6.9%
A4	76	57%±6.7%
R1601	93	61%±5.1%
ATCC15834	91	53%±5.8%

Value are mean ± SD (n=3). The data were obtained as a mean of three replications. 33 explants were used in each test, and a total of 99 explants were used. After removing the explants of bacterial , the total number of rest explants were showed in the second column. The induction efficiency was counted as the proportion of explants induced hairy roots to total explants 20 days after infection.

Table S2 List of the primers used in this study.

**Gene name**	**Primer sequence**
MtPDS-tRNA-CRISPR-35S-B-1	TAGGTCTCAGTCAAACAAAGCACCAGTG
MtPDS-tRNA-CRISPR-sgRNA-2	GCGGTCTCAGGCTTGTGACCATGCACCAGCCGGGAA
MtPDS-tRNA-CRISPR-sgRNA-3	TAGGTCTCAAGCCTATATTGCGTTTTAGAGCTAGAAA
MtPDS-tRNA-CRISPR-35S-B-4	TAGGTCTCAAAACAAAAAAAGCACCGACTCGGTGCC
bar-F	AGTCGACCGTGTACGTCTCC

bar-R	GAAGTCCAGCTGCCAGAAAC
rolB-F	AAGTGCTGAAGGAACAATC
rolB-R	CAAGTGAATGAACAAGGAAC
Cas9-F	ATCCAAGCGAAACGGGAGTT
Cas9-R	ACCGCCACTCCATCAAGAAG
virG-F	CCTTGGGCGTCGTCATAC
virG-R	TCGTCCTCGGTCGTTTCC
MtPDS-F	GATTATCCACGTCCTGAGCT
MtPDS-R	TGTAGGCCGGTCTCATACCA
Cas9-qRT-F	GCCGCTCTGCTTATCCCT
Cas9-qRT-R	ACATCGTCCTCACTCTTACTCTCTT
MtActin-qRT-F	CCACATGCCATCCTTCGTTT
MtActin-qRT-R	TGTCACGGACAATTTCCCG

Red letters and dark blue letters indicate the recognition sequences and cutting sites of *Bsa* I, respectively; light blue letters indicate the target sequences in *MtPDS*.

Table S3 The sequence of pYLCRISPR/Cas9 35S-B-AtU3b

tggcaggatatattgtggtgtaaacaaattgacgcttagacaacttaataacacattgcggacgtttttaatgtactgaattaacgccgaattaattc
gggggatctggattttagtactggattttggttttaggaattagaaattttattgatagaagtattttacaaatacaaatacatactaagggtttcttata
tgctcaacacatgagcgaaaccctataggaaccctaattcccttatctgggaactactcacacattattatggagaaactcgagtcaaatctcggt
gacgggcaggaccggacggggcggtaccggcaggctgaagtccagctgccagaaacccacgtcatgccagttcccgtgcttgaagccgg
ccgcccgcagcatgccgcgggggcatatccgagcgcctcgtgcatgcgcacgctcgggtcgttgggcagcccgatgacagcgaccacg
ctcttgaagccctgtgcctccagggacttcagcaggtgggtgtagagcgtggagcccagtcccgtccgctggtggcggggggagacgtaca
cggtcgactcggccgtccagtcgtaggcgttgcgtgccttccaggggcccgcgtaggcgatgccggcgacctcgccgtccacctcggcga
cgagccagggatagcgctcccgcagacggacgaggtcgtccgtccactcctgcggttcctgcggctcggtacggaagttgaccgtgcttgt
ctcgatgtagtggttgacgatggtgcagaccgccggcatgtccgcctcggtggcacggcggatgtcggccgggcgtcgttctgggctcatg
gtagactcgagagagatagatttgtagagagagactggtgatttcagcgtgtcctctccaaatgaaatgaacttccttatatagaggaaggtctt
gcgaaggatagtgggattgtgcgtcatcccttacgtcagtggagatatcacatcaatccacttgctttgaagacgtggttggaacgtcttcttttc
cacgatgctcctcgtgggtgggggtccatctttgggaccactgtcggcagaggcatcttgaacgatagcctttcctttatcgcaatgatggcattt
gtaggtgccaccttccttttctactgtccttttgatgaagtgacagatagctgggcaatggaatccgaggaggtttcccgatattaccctttgttgaa
aagtctcaatagccctttggtcttctgagactgtatctttgatattcttggagtagacgagagtgtcgtgctccaccatgttatcacatcaatccactt
gctttgaagacgtggttggaacgtcttctttttccacgatgctcctcgtgggtgggggtccatctttgggaccactgtcggcagaggcatcttgaa
cgatagcctttcctttatcgcaatgatggcatttgtaggtgccaccttccttttctactgtccttttgatgaagtgacagatagctgggcaatggaatc

cgaggaggtttcccgatattaccctttgttgaaaagtctcaatagccctttggtcttctgagactgtatctttgatattcttggagtagacgagagtgt
cgtgctccaccatgttggcaagctgctctagccaatacgcaaaccgcctctccccgcgcgttggccgattcattaatgcagctggcacgacag
gtttcccgactggaaagcgggcagtgagcgcaacgcaattaatgtgagttagctcactcattaggcaccccaggctttacactttatgcttccgg
ctcgtatgttgtgtggaattgtgagcggataacaatttcacacaggaaacagctatgaccatgattacgaattcgagctcggtaccaactataac
ggtcctaaggtagcgaaggatccgctcgctaccttaagagaggatatccctccatcctataatgtaggctataggaactagggcaaggccgg
ccatgcggccgcgtcactggattttggttttaggaattagaaattttattgatagaagtattttacaaatacaaatacatactaagggtttcttatatgc
tcaacacatgagcgaaaccctataagaaccctaattcccttatctgggaactactcacacattattctggagaaaaatagagagagatagatttgt
agagagagactggtgatttttgcggactctagatcaggtggatcctacttctttttcttagcctgtccggcctttttggtggcagcaggacgcttatc
accaccaagctgggaaaggtcgatacgagtctcgtaaagaccggtgatggactggtggatgagagtagcgtcgagaacctccttggtggac
gtgtaacgcttcctgtcgatggtggtgtcgaagtacttgaaagcagcaggggcgccgaggttcgtgagcgtgaagaggtggatgatgttctcg
gcctgctcgcggatgggcttgtcgcggtgcttgttgtaggcggagaggaccttgtcgaggttagcgtcagcgaggatgacgcgcttggagaa
ctcggagatctgctcgatgatctcgtcgaggtagtgcttgtgctgctccacgaagagctgcttctgctcgttatcctcaggggaacccttgagctt
ctcgtagtgggaggcgaggtagaggaagttcacgtacttggaaggaagagcaagctcgttacccttctggagctcaccagcggaagccagc
atcctcttacgaccgttctcgagctcgaaaagagagtacttggggagcttgatgatgaggtccttcttgacctccttgtagcccttggcctcgag
gaagtcgatcgggttcttctcgaaggaggagcgctccatgatggtgatgccgaggagctccttgacggacttgagcttcttcgacttacccttct
ccaccttggcgaccacgaggacggagtaggcgacagtaggggagtcgaaaccaccgtacttcttagggtcccaatccttcttcctagcgatg
agcttgtccgagtttctctttggaaggatagactccttggagaagccaccggtctggacctcggtcttcttgacgatgttaacctgaggcataga
aaggaccttgcgaacagtagcgaagtcgcgacccttgtcccaaacgatctcaccagtctcaccgttcgtctcgataagagggcgcttgcggat
ctcgccgttggcgagggtgatctcggtcttgaagaagttcatgatgttggagtagaagaagtacttggcggtggccttgccgatctcctgctcg
gacttggcgatcatcttacgaacgtcgtagaccttgtagtcaccgtagacgaactcggactcaagcttagggtacttcttgataagagcggtacc
aacgacagcgttaaggtaagcatcgtgagcgtggtggtagttgttgatctcgcggaccttgtagaactggaagtccttgcggaagtcggagac
gagcttggacttgagggtgatcaccttgacctcgcggatgagcttgtcgttctcgtcgtacttggtgttcatcctagaatcgaggatctgagcaac
gtgcttggtaatctgcctcgtctcaacaagctgcctcttgatgaaaccagccttgtcaagctcggaaaggccacccctctcagccttcgtgaggt
tgtcgaacttcctctgggtaatgagcttagcgttgagaagctgcctccagtagttcttcatcttcttgacaacctcctcggaagggacgttgtccg
acttacccctgttcttgtcggacctcgtgaggaccttgttgtcgatggagtcatccttaaggaaagactgaggaacaatgtggtcgacgtcgtag
tcagaaagcctgttgatgtcgagctcctggtcaacgtacatatccctaccgttctggaggtagtagaggtagagcttctcgttctggagctgggt
gttctcgacagggtgctccttaaggatctgagaaccaagctccttgataccctcctcaatcctcttcatgcgctccctcgagttcttctgacccttct
gggtagtctggttctcacgagccatctcgatgacgatgttctcaggcttgtgacgacccataaccttgacaagctcatcgacaaccttaacagtc
tgaaggatgcccttcttgatagcaggggaaccagcaaggttagcaatgtgctcgtggagagagtcgccctgaccggacacctgagccttctg
aatatcctccttgaaggtaagagagtcatcgtggatgagctgcatgaagttcctgttagcgaaaccatcagacttgaggaagtcgaggatagtc
ttgccgctctgcttatccctgataccgttgatgagcttgcgggagagcctaccccaaccggtgtaacggcgacgcttgagctgcttcataacctt
gtcatcgaagagatgagcgtaagtcttgagcctctcctcgatcatctccctatcctcgaagagagtaagagtgaggacgatgtcctcgaggatg
tcctcgttctcctcgttgtcgaggaagtccttgtccttgatgatcttgaggagatcgtggtaggtaccgagagaagcgttgaaacggtcctcaac
gccgctgatctcgacggagtcgaagcactcgatcttcttgaagtagtcctccttgagctgcttgacggtgaccttgcggttggtcttgaagagga
ggtcaacgatagccttcttctgctcgccggagaggaaggcaggcttgcgcataccctcggtgacgtacttgaccttggtgagctcgttgtagac
cgtgaagtactcgtagaggagggagtgcttggggaggaccttctcgttggggaggttcttgtcgaagttggtcatgcgctcgatgaaggactg
ggcggaagcacccttgtcaacaacctcctcgaagttccaaggggtgatagtctcctcggacttcctagtcatccaagcgaaacgggagttacc
acgagcaagaggaccaacgtagtaaggaatacggaaagtaaggatcttctcgatcttctcacggttgtccttgaggaaagggtagaagtcctc
ctgcctacgaaggatggcgtgaagctcaccaaggtggatctggtgagggatagagccgttatcgaaagtcctctgcttcctaaggaggtcctc
acggttaagcttaacgagaagctcctcagtaccatccatcttctcaaggattggcttgatgaacttgtagaactcctcctggctagctccaccgtc
aatgtaaccggcgtagccgttcttggactggtcgaagaagatctccttgtacttctcggggagctgctgacgaacaagagccttgaggagggt

gaggtcctggtggtgctcgtcgtagcgcttgatcatggaagcagaaagaggagccttagtgatctcagtgttaaccctaaggatatccgaaag
gaggatagcatcggagaggttcttagcagcaaggaagagatcagcgtactgatctccaatctgagcgaggaggttgtcgagatcatcgtcgt
aggtatccttggagagctgaagcttagcgtcctcagcgaggtcgaagttggacttgaagttaggggtcagaccgagggagagagcgatgag
gttaccgaaaagaccgttcttcttctcaccagggagctgggcgatgaggttctcgagacgcctggacttggagagcctagcggaaaggatcg
ccttagcgtcgacaccggaagcgttgatagggttctcctcgaagagctggttgtaggtctggacgagctggatgaagagcttgtccacgtcgg
agttatcagggttaaggtcaccctcgataaggaagtgaccacggaacttgatcatgtgagcgagagcaaggtagatgagacgaagatcagc
cttatcagtagagtcaacaagcttcttacgaaggtggtagatagtggggtacttctcgtggtaggcgacctcgtcgacgatgttgccgaagatg
gggtggcgctcgtgcttcttgtcctcctccacgaggaaggactcctcgaggcggtggaagaaggagtcgtcgaccttcgccatctcgttgga
gaagatctcctggaggtagcagatgcggttcttgcggcgggtgtagcggcggcgggcggtgcgcttgaggcgggtcgcctccgccgtctc
gccggagtcgaagaggagggcgccgatgaggttcttcttgatggagtggcggtcggtgttgcccaggaccttgaacttcttggacgggacct
tgtactcgtcggtgatcaccgcccagccgacgctgttggtgccgatgtcgaggccgatggagtacttcttgtcagccgcaggcaccccgtga
ataccaaccttccgcttcttcttaggagccatggctatcgttcgtaaatggtgaaaattttcagaaaattgcttttgctttaaaagaaatgatttaaatt
gctgcaatagaagtagaatgcttgattgcttgagattcgtttgttttgtatatgttgtgttgagaactctcgacgtcctctccaaatgaaatgaacttc
cttatatagaggaagggtcttgcgaaggatagtgggattgtgcgtcatcccttacgtcagtggagatatcacatcaatccacttgctttgaagac
gtggttggaacgtcttctttttccacgatgctcctcgtgggtgggggtccatctttgggaccactgtcggcagaggcatcttcaacgatggccttt
cctttatcgcaatgatggcatttgtaggagccaccttccttttccactatcttcacaataaagtgacagatagctgggcaatggaatccgaggagg
tttccggatattaccctttgttgaaaagtctcaattgccctttggtcttctgagactgtatctttgatatttttggagtagacaagtgtgtcgtgctccac
catgttatcacatcaatccacttgctttgaagacgtggttggaacgtcttctttttccacgatgctcctcgtgggtgggggtccatctttgggaccac
tgtcggcagaggcatcttcaacgatggcctttcctttatcgcaatgatggcatttgtaggagccaccttccttttccactatcttcacaataaagtga
cagatagctgggcaatggaatccgaggaggtttccggatattaccctttgttgaaaagtctcaattgccctttggtcttctgagactgtatctttgat
atttttggagtagacaagtgtgtcgtgctccacAtgttgaccggtaaggcctccgttttacctgtggaatcggcagcaaagg
aTTTACTTTAAATTTTTTCTTATGCAGCCTGTGATGGATACCTGAATCAAACAAATGGCGTCTGGGTTT
AAGAAGATCTGTTTTGGCTATGTTGGACGAAACAAGTGAACTTTTAGGATCAACTTCAGTTTATATATG
GAGCTTATATCGAGCAATAAGATAAGTGGGCTTTTTATGTAATTTAATGGGCTATCGTCCATAGATTCA
CTAATACCCATGCCCAGTACCCATGTATGCGTTTCATATAAGCTCCTAATTTCTCCCACATCGCTCAAA
TCTAAACAAATCTTGTTGTATATATAACACTGAGGGAGCAACATTGGTCAggagaccgaggtctcggttttagag
ctagaaatagcaagttaaaataaggctagtccgttatcaacttgaaaaagtggcaccgagtcggtgctttttcaagagcttggagtgga
tggacctctcgagctagcggccgcatgcatcgatctcctacatcgtataaattagcctatacgaagttattgcatctatgtcgggtgcg
gagaaagaggtaatgaaatggcagtattagatctgataacttcgtataatgtatgctatacgaagttatgactgcaggtcgacacccataatagct
gtttgccaagcttggcactggccgtcgtttttacaacgtcgtgactgggaaaaccctggcgttacccaacttaatcgccttgcagcacatccccc
tttcgccagctggcgtaatagcgaagaggcccgcaccgatcgcccttcccaacagttgcgcagcctgaatggcgaatgctagagcagcttga
gcttggatcagattgtcgtttcccgccttcagtttaaactatcagtgtttgacaggatatattggcgggtaaacctaagagaaaagagcgtttatta
gaataacggatatttaaaagggcgtgaaaaggtttatccgttcgtccatttgtatgtgcatgccaaccacagggttcccctcgggatcaaagtac
tttgatccaacccctccgctgctatagtgcagtcggcttctgacgttcagtgcagccgtcttctgaaaacgacatgtcgcacaagtcctaagttac
gcgacaggctgccgccctgcccttttcctggcgttttcttgtcgcgtgttttagtcgcataaagtagaatacttgcgactagaaccggagacatta
cgccatgaacaagagcgccgccgctggcctgctgggctatgcccgcgtcagcaccgacgaccaggacttgaccaaccaacgggccgaac
tgcacgcggccggctgcaccaagctgttttccgagaagatcaccggcaccaggcgcgaccgcccggagctggccaggatgcttgaccacc
tacgccctggcgacgttgtgacagtgaccaggctagaccgcctggcccgcagcacccgcgacctactggacattgccgagcgcatccagg
aggccggcgcgggcctgcgtagcctggcagagccgtgggccgacaccaccacgccggccggccgcatggtgttgaccgtgttcgccgg
cattgccgagttcgagcgttccctaatcatcgaccgcacccggagcgggcgcgaggccgccaaggcccgaggcgtgaagtttggcccccg
ccctaccctcaccccggcacagatcgcgcacgcccgcgagctgatcgaccaggaaggccgcaccgtgaaagaggcggctgcactgcttg

gcgtgcatcgctcgaccctgtaccgcgcacttgagcgcagcgaggaagtgacgcccaccgaggccaggcggcgcggtgccttccgtgag
gacgcattgaccgaggccgacgccctggcggccgccgagaatgaacgccaagaggaacaagcatgaaaccgcaccaggacggccagg
acgaaccgtttttcattaccgaagagatcgaggcggagatgatcgcggccgggtacgtgttcgagccgcccgcgcacgtctcaaccgtgcg
gctgcatgaaatcctggccggtttgtctgatgccaagctggcggcctggccggccagcttggccgctgaagaaaccgagcgccgccgtcta
aaaaggtgatgtgtatttgagtaaaacagcttgcgtcatgcggtcgctgcgtatatgatgcgatgagtaaataaacaaatacgcaaggggaac
gcatgaaggttatcgctgtacttaaccagaaaggcgggtcaggcaagacgaccatcgcaacccatctagcccgcgccctgcaactcgccgg
ggccgatgttctgttagtcgattccgatccccagggcagtgcccgcgattgggcggccgtgcgggaagatcaaccgctaaccgttgtcggca
tcgaccgcccgacgattgaccgcgacgtgaaggccatcggccggcgcgacttcgtagtgatcgacggagcgccccaggcggcggacttg
gctgtgtccgcgatcaaggcagccgacttcgtgctgattccggtgcagccaagcccttacgacatatgggccaccgccgacctggtggagct
ggttaagcagcgcattgaggtcacggatggaaggctacaagcggcctttgtcgtgtcgcgggcgatcaaaggcacgcgcatcggcggtga
ggttgccgaggcgctggccgggtacgagctgcccattcttgagtcccgtatcacgcagcgcgtgagctacccaggcactgccgccgccgg
cacaaccgttcttgaatcagaacccgagggcgacgctgcccgcgaggtccaggcgctggccgctgaaattaaatcaaaactcatttgagtta
atgaggtaaagagaaaatgagcaaaagcacaaacacgctaagtgccggccgtccgagcgcacgcagcagcaaggctgcaacgttggcca
gcctggcagacacgccagccatgaagcgggtcaactttcagttgccggcggaggatcacaccaagctgaagatgtacgcggtacgccaag
gcaagaccattaccgagctgctatctgaatacatcgcgcagctaccagagtaaatgagcaaatgaataaatgagtagatgaattttagcggcta
aaggaggcggcatggaaaatcaagaacaaccaggcaccgacgccgtggaatgccccatgtgtggaggaacgggcggttggccaggcgt
aagcggctgggttgtctgccggccctgcaatggcactggaacccccaagcccgaggaatcggcgtgacggtcgcaaaccatccggcccg
gtacaaatcggcgcggcgctgggtgatgacctggtggagaagttgaaggccgcgcaggccgcccagcggcaacgcatcgaggcagaag
cacgccccggtgaatcgtggcaagcggccgctgatcgaatccgcaaagaatcccggcaaccgccggcagccggtgcgccgtcgattagg
aagccgcccaagggcgacgagcaaccagatttttttcgttccgatgctctatgacgtgggcacccgcgatagtcgcagcatcatggacgtggc
cgttttccgtctgtcgaagcgtgaccgacgagctggcgaggtgatccgctacgagcttccagacgggcacgtagaggtttccgcagggccg
gccggcatggccagtgtgtgggattacgacctggtactgatggcggtttcccatctaaccgaatccatgaaccgataccgggaagggaagg
gagacaagcccggccgcgtgttccgtccacacgttgcggacgtactcaagttctgccggcgagccgatggcggaaagcagaaagacgac
ctggtagaaacctgcattcggttaaacaccacgcacgttgccatgcagcgtacgaagaaggccaagaacggccgcctggtgacggtatccg
agggtgaagccttgattagccgctacaagatcgtaaagagcgaaaccgggcggccggagtacatcgagatcgagctagctgattggatgta
ccgcgagatcacagaaggcaagaacccggacgtgctgacggttcaccccgattactttttgatcgatcccggcatcggccgttttctctaccgc
ctggcacgccgcgccgcaggcaaggcagaagccagatggttgttcaagacgatctacgaacgcagtggcagcgccggagagttcaagaa
gttctgtttcaccgtgcgcaagctgatcgggtcaaatgacctgccggagtacgatttgaaggaggaggcggggcaggctggcccgatcctag
tcatgcgctaccgcaacctgatcgagggcgaagcatccgccggttcctaatgtacggagcagatgctagggcaaattgccctagcagggga
aaaaggtcgaaaaggtctgtttcctgtggatagcacgtacattgggaacccaaagccgtacattgggaaccggaacccgtacattgggaacc
caaagccgtacattgggaaccggtcacacatgtaagtgactgatataaaagagaaaaaaggcgatttttccgcctaaaactctttaaaacttatt
aaaactcttaaaacccgcctggcctgtgcataactgtctggccagcgcacagccgaagagctgcaaaaagcgcctacccttcggtcgctgcg
ctccctacgccccgccgcttcgcgtcggcctatcgcggccgctggccgctcaaaaatggctggcctacggccaggcaatctaccagggcgc
ggacaagccgcgccgtcgccactcgaccgccggcgcccacatcaaggcaccctgcctcgcgcgtttcggtgatgacggtgaaaacctctg
acacatgcagctcccggagacggtcacagcttgtctgtaagcggatgccgggagcagacaagcccgtcagggcgcgtcagcgggtgttgg
cgggtgtcggggcgcagccatgacccagtcacgtagcgatagcggagtgtatactggcttaactatgcggcatcagagcagattgtactgag
agtgcaccatatgcggtgtgaaataccgcacagatgcgtaaggagaaaataccgcatcaggcgctcttccgcttcctcgctcactgactcgct
gcgctcggtcgttcggctgcggcgagcggtatcagctcactcaaaggcggtaatacggttatccacagaatcaggggataacgcaggaaag
aacatgtgagcaaaaggccagcaaaaggccaggaaccgtaaaaaggccgcgttgctggcgtttttccataggctccgcccccctgacgagc
atcacaaaaatcgacgctcaagtcagaggtggcgaaacccgacaggactataaagataccaggcgtttccccctggaagctccctcgtgcg
ctctcctgttccgaccctgccgcttaccggatacctgtccgcctttctcccttcgggaagcgtggcgctttctcatagctcacgctgtaggtatctc

agttcggtgtaggtcgttcgctccaagctgggctgtgtgcacgaaccccccgttcagcccgaccgctgcgccttatccggtaactatcgtcttg
agtccaacccggtaagacacgacttatcgccactggcagcagccactggtaacaggattagcagagcgaggtatgtaggcggtgctacaga
gttcttgaagtggtggcctaactacggctacactagaaggacagtatttggtatctgcgctctgctgaagccagttaccttcggaaaaagagttg
gtagctcttgatccggcaaacaaaccaccgctggtagcggtggtttttttgtttgcaagcagcagattacgcgcagaaaaaaaggatctcaaga
agatcctttgatcttttctacggggtctgacgctcagtggaacgaaaactcacgttaagggattttggtcatgcattctaggtactaaaacaattcat
ccagtaaaatataatattttattttctcccaatcaggcttgatccccagtaagtcaaaaaatagctcgacatactgttcttccccgatatcctccctga
tcgaccggacgcagaaggcaatgtcataccacttgtccgccctgccgcttctcccaagatcaataaagccacttactttgccatctttcacaaag
atgttgctgtctcccaggtcgccgtgggaaaagacaagttcctcttcgggcttttccgtctttaaaaaatcatacagctcgcgcggatctttaaatg
gagtgtcttcttcccagttttcgcaatccacatcggccagatcgttattcagtaagtaatccaattcggctaagcggctgtctaagctattcgtatag
ggacaatccgatatgtcgatggagtgaaagagcctgatgcactccgcatacagctcgataatcttttcagggctttgttcatcttcatactcttccg
agcaaaggacgccatcggcctcactcatgagcagattgctccagccatcatgccgttcaaagtgcaggacctttggaacaggcagctttcctt
ccagccatagcatcatgtccttttcccgttccacatcataggtggtccctttataccggctgtccgtcatttttaaatataggttttcattttctcccacc
agcttatataccttagcaggagacattccttccgtatcttttacgcagcggtatttttcgatcagtttttcaattccggtgatattctcattttagccattt
attatttccttcctcttttctacagtatttaaagataccccaagaagctaattataacaagacgaactccaattcactgttccttgcattctaaaacctta
aataccagaaaacagctttttcaaagttgttttcaaagttggcgtataacatagtatcgacggagccgattttgaaaccgcggtgatcacaggca
gcaacgctctgtcatcgttacaatcaacatgctaccctccgcgagatcatccgtgtttcaaacccggcagcttagttgccgttcttccgaatagca
tcggtaacatgagcaaagtctgccgccttacaacggctctcccgctgacgccgtcccggactgatgggctgcctgtatcgagtggtgattttgt
gccgagctgccggtcggggagctgttggctggctgg

AtU3b Promoter

The two BsaI cutting sites

SgRNA sequence
